# Supplementary material for: Synergistic Effects of Probiotics and Phytobiotics on the Intestinal Microbiota in Young Broiler Chicken
Source: Microorganisms. 2019 Dec 11;7(12):684. doi: 10.3390/microorganisms7120684 (PMC6956037; doi:10.3390/microorganisms7120684)
Supplement: Supplementary file 1 [file microorganisms-07-00684-s001.pdf]

Table S1: Primer sequences and annealing temperatures for the detection of bacterial groups and species in gut intestine

**Target item**	**Primers**	**Sequence**	**Annealing T (°C)**
Ccoccoides-Cluster	Forward	AAATGACGGTACCTGACTAA	60
(Cluster XIVa)	Reverse	CTTTGAGTTTCATTCTTGCGAA	
C.leptum-Cluster	Forward	GCACAAGCAGTGGAGT	60
(Cluster IV)	Reverse	CTTCCTCCGTTTTGTCAA	
Clostridien-Cluster I	Forward	TACCHRAGGAGGAAGCCAC	63
	Reverse	GTTCTTCCTAATCTCTACGCAT	
Clostridium perfringens group	Forward	ATGCAAGTCGAGCGA(G/T)G	55
	Reverse	TATGCGGTATTAATCT(C/T)CCTTT	
L. acidophilus	Forward	AGCTGAACCAACAGATTCAC	55
	Reverse	ACTACCAGGGTATCTAATCC	
L. reuteri	Forward	CCCAACTGATTGATGGTGCT	55
	Reverse	GGGCAGGTTACCTACGTGTT	
L. salivarius	Forward	CGAAACTTTCTTACACCGAATGC	58
	Reverse	TCCCCTTCTTTTAGGCAGGTTA	
L. agilis	Forward	GGCGAACGGGTGAGTAACA	60
	Reverse	CCAAAAGTGATAGCCGAAACCA	60
Bact—*Prev* —*Porphyromonas* (BPP)	Forward	GGTGTCGGCTTAAGTGCCAT	55
	Reverse	CGGAYGTAAGGGCCGTGC	
Enterobacteriaceae	Forward	GTBTCDCCRCGCAGRC	55
	Reverse	TGCGYCTGGTRATCTA	

Escherichia group	Forward	GTTAATACCTTTGCTCATTGA	55
	Reverse	ACCAGGGTATCTAATCCTGTT	
Lactobacillus spp.	Forward	AGCAGTAGGGAATCTTCCA	58
	Reverse	CACCGCTACACATGGAG	
Integrase 1	Forward	AGGATGCGAACCACTTCATC	55
	Reverse	GCTGTTCTTCTACGGCAAGG	55.2

Table S2: Relative abundance [%] of bacterial phyla in the crop of 13-day-old broiler chicken fed different probiotics and phytobiotics

	Control	LS1	LA73	Formulation C	Formulation L	LS1 & Formulation C	LS1 & Formulation L	LA73 & Formulation C	LA73 & Formulation L	Pooled SEM	p-value[1]
Firmicutes	99.1	98.7	99.4	99.4	98.7	99.5	99.8	99.7	99.9	0.13	0.346
Proteobacteria	0.58	0.89	0.33	0.31	0.81	0.25	0.17	0.11	0.07	0.09	0.397
Bacteroidetes	0.25	0.29	0.18	0.19	0.37	0.13	0.05	0.19	0.03	0.04	0.327
Actinobacteria	0.03	0.07	0.10	0.09	0.02	0.04	0.01	0.05	0.04	0.01	0.650
Epsilonbacteraeota	0.03	n.d.	0.04	0.03	0.11	0.11	n.d.	n.d.	n.d.	0.01	0.117

n.d. = not detected

[1] = Kruskal-Wallis Test

Table S3: Relative abundance [%] of bacterial phyla in the caecum of 13-day-old broiler chicken fed different probiotics and phytobiotics

	Control	LS1	LA73	Formulation C	Formulation L	LS1 & Formulation C	LS1 & Formulation L	LA73 & Formulation C	LA73 & Formulation L	Pooled SEM	p-value[1]
Firmicutes	75.4	76.6	78.5	77.1	80.0	79.2	80.3	77.6	78.3	0.86	0.946
Bacteroidetes	24.4	23.1	21.2	22.5	19.6	20.3	19.3	22.1	21.4	0.86	0.946
Tenericutes	0.14	0.22	0.12	0.16	0.19	0.38	0.26	0.10	0.13	0.04	0.905
Actinobacteria	0.07	0.08	0.05	0.13	0.11	0.04	0.07	0.06	0.05	0.01	0.229
Proteobacteria	0.04	0.01	0.04	0.003	0.04	0.002	0.002	0.01	0.02	0.01	0.804

[1] = Kruskal-Wallis Test

Table S4: Relative abundance [%] of dominant bacterial order in the crop of 13-day-old broiler chicken fed different probiotics and phytobiotics

	Control	LS1	LA73	Formulation C	Formulation L	LS1 & Formulation C	LS1 & Formulation L	LA73 & Formulation C	LA73 & Formulation L	Pooled SEM	p-value[1]
Lactobacillales	$98.8^{a,b}$	98.2^{a}	98.9^{a}	97.8^{a}	98.1^{a}	99.3^{b}	99.7^{b}	$99.1^{a,b}$	99.7^{b}	0.192	**0.024**
Clostridiales	0.27^{b}	0.52^{c}	0.40^{c}	1.58^{d}	0.57^{c}	0.12^{b}	0.02^{a}	0.53^{c}	0.16^{b}	0.123	**0.019**
Pseudomonadales	0.21	0.36	0.05	0.01	0.26	0.02	0.04	0.01	0.02	0.04	0.21
Enterobacteriales	0.03	0.03	0.04	0.10	n.d.	0.02	0.02	0.05	0.02	0.008	0.113
Aeromonadales	0.28	0.25	0.17	0.15	0.27	0.20	0.07	0.03	n.d.	0.033	0.192
Bacteroidales	0.23	0.28	0.16	0.16	0.31	0.10	0.04	0.18	0.02	0.037	0.611
Betaproteobacteriales	0.04	$0.24^{b,c}$	0.06^{b}	$0.04^{a.b}$	0.25^{c}	0.01^{a}	$0.04^{a.b}$	0.01^{a}	n.d.	0.03	**0.029**
Micrococcales	0.03	0.06	0.09	0.07	0.02	0.04	0.01	0.05	0.04	0.011	0.864
Campylobacterales	0.03	n.d.	0.04	0.03	0.11	0.11	n.d.	n.d.	n.d.	0.014	0.117
Bacillales	0.01	n.d.	0.04	0.01	0.01	0.03	0.01	n.d.	n.d.	0.006	0.907
Flavobacteriales	0.02	0.01	0.02	0.02	0.06	0.03	0.02	0.01	n.d.	0.005	0.621

n.d. = not detected

[1] = Kruskal-Wallis Test; superscripts denote significant differences within a row (Mann-Whitney U Test, $p \leq 0.05$)

Table S5: Relative abundance [%] of dominant bacterial order in the caecum of 13-day-old broiler chicken fed different probiotics and phytobiotics

	Control	LS1	LA73	Formulation C	Formulation L	LS1 & Formulation C	LS1 & Formulation L	LA73 & Formulation C	LA73 & Formulation L	Pooled SEM	p-value[1]
Clostridiales	65.9	68.1	66.7	67.2	65.9	71.7	67.4	68.9	70.5	1.053	0.926
Bacteroidales	24.3	23.0	21.2	22.5	19.6	20.3	19.3	22.1	21.4	0.859	0.946
Lactobacillales	7.2	6.0	9.1	7.5	11.0	5.9	10.6	7.0	5.6	0.656	0.343
Erysipelotrichales	1.4	1.5	1.8	1.5	1.3	1.0	1.2	0.79	1.6	0.191	0.711
Bacillales	0.68	0.96	0.81	0.75	1.5	0.49	0.96	0.87	0.57	0.139	0.900
Unidentified *Mollicutes*	0.12	0.22	0.12	0.12	0.19	0.36	0.24	0.08	0.03	0.039	0.482
Coriobacteriales	0.07	0.08	0.05	0.13	0.11	0.04	0.07	0.06	0.05	0.008	0.229
Unidentified *Firmicutes*	0.05	n.d.	n.d.	0.04	n.d.	0.05	0.08	0.06	0.01	0.011	0.417

Anaeroplasmatales	0.02	n.d.	n.d.	0.03	0.01	0.03	0.03	0.03	0.10	0.009	0.560
Enterobacteriales	0.04	0.01	0.04	n.d.	0.04	n.d.	n.d.	0.01	0.02	0.007	0.480

n.d. = not detected

[1] = Kruskal-Wallis Test

Table S6: Relative abundance [%] of dominant bacterial genera in the crop of 13-day-old broiler chicken fed different probiotics and phytobiotics

	Control	LS1	LA73	Formulation C	Formulation L	LS1 & Formulation C	LS1 & Formulation L	LA73 & Formulation C	LA73 & Formulation L	Pooled SEM	p-value[1]
Lactobacillus	98.9$^{a.b}$	98.3^{a}	99.0$^{a.b}$	98.0^{a}	98.2^{a}	99.4$^{a.b}$	99.8^{b}	99.2$^{a.b}$	99.7^{b}	0.181	**0.013**
Acinetobacter	0.21	0.32	0.04	n.d.	0.24	0.02	0.04	0.01	0.01	0.036	0.143
Aeromonas	0.28	0.25	0.17	0.15	0.27	0.20	0.07	0.03	n.d.	0.033	0.192
Bacteroides	0.21	0.20	0.14	0.16	0.26	0.08	0.03	0.12	0.02	0.033	0.594
Clostridium_sensu_stricto_1	0.11$^{a.b}$	0.24^{b}	0.29^{b}	0.04^{a}	0.18$^{a.b}$	0.06^{a}	0.02^{a}	0.18$^{a.b}$	n.d.	0.029	**0.045**
Unidentified *Clostridiales*	0.08	0.16	0.02	0.68	0.18	0.03	n.d.	0.17	0.11	0.057	0.329
Blautia	0.06	0.02	0.02	0.16	0.02	0.01	n.d.	0.06	0.01	0.015	0.504
Cloacibacterium	0.01	0.01	0.01	0.02	0.06	0.03	0.01	0.01	n.d.	0.005	0.36
Faecalibacterium	0.01	0.03	0.07	0.38	0.15	n.d.	n.d.	0.03	0.01	0.035	0.175
Pantoea	0.03^{a}	0.03^{a}	0.02^{a}	0.07^{b}	n.d.	n.d.	0.01^{a}	0.02^{a}	0.01^{a}	0.006	**0.027**
Arcobacter	0.03	n.d.	0.04	0.03	0.11	0.11	n.d.	n.d.	n.d.	0.015	0.117
Comamonas	n.d.	0.20	0.02	0.01	0.13	n.d.	0.01	n.d.	n.d.	0.025	0.214
Plantibacter	0.02	0.03	0.09	n.d.	n.d.	0.02	n.d.	n.d.	0.01	0.01	0.553
Macellibacteroides	n.d.	0.02	0.02	0.01	0.03	0.01	n.d.	0.05	n.d.	0.005	0.656
Azospira	0.02	0.03	0.01	0.01	n.d.	n.d.	0.01	n.d.	n.d.	0.003	0.738
Escherichia/Shigella	n.d.	n.d.	0.03	0.02	n.d.	n.d.	n.d.	0.02	0.01	0.004	0.575
Zoogloea	n.d.	0.01	0.01	0.01	0.09	n.d.	0.01	n.d.	n.d.	0.008	0.208
Microbacterium	n.d.	0.03	n.d.	0.04	n.d.	n.d.	0.01	0.03	0.03	0.006	0.464
Macrococcus	0.01	n.d.	n.d.	n.d.	0.01	0.02	0.01	n.d.	n.d.	0.002	0.671
Rothia	n.d.	n.d.	n.d.	0.01	0.01	0.02	n.d.	0.02	n.d.	0.002	0.453
Acetobacteroides	0.03	0.06	n.d.	n.d.	0.02	0.01	n.d.	n.d.	n.d.	0.007	0.54
Stenotrophomonas	n.d.	n.d.	n.d.	n.d.	0.01	n.d.	0.01	0.01	0.02	0.002	0.439

n.d. = not detected

[1] = Kruskal-Wallis Test; superscripts denote significant differences within a row (Mann-Whitney U Test, $p \leq 0.05$)

Table S7: Relative abundance [%] of dominant bacterial genera in the caecum of 13-day-old broiler chicken fed different probiotics and phytobiotics

	Control	LS1	LA73	Formulation C	Formulation L	LS1 & Formulation C	LS1 & Formulation L	LA73 & Formulation C	LA73 & Formulation L	Pooled SEM	p-value[1]
Unidentified *Clostridiales*	$28.4^{A,B}$	$35.6^{B,C}$	28.4^{B}	$24.9^{A,B}$	$26.2^{A,B}$	22.7^{A}	$30.0^{B,C}$	23.2^{A}	35.4^{C}	1.256	0.087
Bacteroides	24.4	23.1	21.3	22.6	19.7	20.3	19.3	22.2	21.5	0.859	0.946
Faecalibacterium	$11.6^{a,b}$	2.7^{a}	$14.0^{a,b}$	$13.4^{a,b}$	$10.5^{a,b}$	22.7^{b}	9.3^{a}	20.0^{b}	7.9 a	1.535	0.035
Lactobacillus	7.2	5.9	9.2	7.6	11.0	5.9	10.6	7.0	5.6	0.651	0.335
Blautia	5.8	5.3	3.9	7.0	6.5	3.8	4.5	4.5	5.5	0.575	0.591
Subdoligranulum	1.6	3.4	2.6	1.0	2.0	5.0	3.7	1.8	3.0	0.323	0.198
Marvinbryantia	2.7	3.1	3.4	4.6	1.7	1.5	1.5	1.8	1.6	0.102	0.508
Eisenbergiella	1.5	1.9	1.7	1.7	1.2	1.5	1.5	1.7	1.7	0.277	0.808
Fusicatenibacter	1.5	1.7	0.9	2.4	1.8	1.2	2.8	1.1	1.4	0.127	0.800
Unidentified *Ruminococcus* (UCG.003)	1.4	2.0	1.2	1.9	1.9	1.1	1.6	1.3	1.7	0.192	0.497
Lachnoclostridium	1.9	1.7	1.5	1.0	1.3	1.3	1.0	1.6	1.3	0.139	0.673
Butyricicoccus	2.3	2.1	0.5	0.5	1.9	0.5	0.5	2.0	1.1	0.076	0.262
Sellimonas	1.9	1.0	0.7	0.3	2.9	1.8	1.2	1.3	0.6	0.038	0.195
Erysipelatoclostridium	1.3	1.5	1.7	1.3	1.4	0.5	1.3	0.7	1.5	0.029	0.670
Unidentified *Bacillales*	0.68	0.95	0.81	0.76	1.57	0.49	0.96	0.87	0.57	0.109	0.900
Anaerostipes	$0.58^{a,b}$	$1.08^{b,c}$	$0.73^{a,b}$	$0.49^{a,b}$	$0.56^{a,b}$	$0.91^{a,b}$	$1.10^{b,c}$	$0.86^{a,b}$	$0.91^{a,b}$	0.05	0.036
Ruminiclostridium_9	0.34	0.38	0.35	0.96	0.98	0.51	0.86	0.44	0.87	0.084	0.192
Negativibacillus	0.42	0.49	0.51	0.5	0.57	0.56	0.68	0.56	0.65	0.019	0.155
Pygmaiobacter	0.35	0.56	0.44	0.42	0.44	0.69	0.72	0.50	0.37	0.109	0.753
Ruminiclostridium_5	0.46	0.36	0.67	0.74	0.78	0.26	0.29	0.36	0.56	0.084	0.544
Flavonifractor	0.21	0.46	0.36	0.52	0.54	0.53	0.45	0.53	0.37	0.067	0.748
Anaerotruncus	0.37	0.20	0.4	0.13	0.16	0.33	0.73	0.31	1.10	0.052	0.854
Unidentified *Clostridiales*	0.41	0.27	0.39	0.34	0.48	0.42	0.42	0.36	0.49	0.035	0.786

Unidentified *Clostridium*	0.14	0.38	0.35	0.5	0.27	0.38	0.31	0.60	0.48	0.011	0.623
Tyzzerella	0.23	0.40	0.6	0.24	0.06	0.21	0.43	0.30	0.13	0.029	0.961
Unidentified *Ruminococcus* (UCG.005)	0.22	0.26	0.12	0.32	0.22	0.41	0.28	0.31	0.29	0.039	0.237
Shuttleworthia	0.26	0.26	0.18	0.28	0.24	0.25	0.27	0.22	0.27	0.007	0.339
Merdibacter	0.06	n.d.	n.d.	0.82	0.02	0.15	0.32	0.29	0.67	0.019	0.852
Unidentified *Firmicutes*	0.27	0.24	0.16	0.16	0.24	0.06	0.17	0.23	0.16	0.016	0.316
Unidentified *Ruminococcus* (UCG.005)	0.24	0.14	0.23	0.2	0.15	0.05	0.33	0.12	0.17	0.011	0.141
Unidentified *Mollicutes* (RF39)	n.d.	0.13	0.21	0.01	0.06	0.46	0.15	0.2	0.18	0.015	0.197
Unidentified *Ruminococcus* (UCG.009)	0.07	0.09	0.18	0.14	0.05	0.21	0.19	0.31	0.27	0.024	0.058
Intestinimonas spp.	0.12	0.22	0.12	0.12	0.19	0.36	0.24	0.08	0.03	0.339	0.126
Unidentified *Lachnospira* (FCS020)	0.08	0.12	0.19	0.13	0.24	0.22	0.13	0.09	0.12	0.002	0.490
Acetitomaculum spp.	0.19	0.03	0.07	0.22	0.03	0.49	n.d.	0.08	0.12	0.007	0.282
Oscillibacter spp.	0.08	0.13	0.09	0.3	0.05	0.06	0.12	0.02	0.11	0.035	0.085
Tyzzerella spp.	0.01	0.02	0.1	0.22	0.11	0.05	0.1	0.05	0.07	0.029	0.240
Unidentified *Clostridium* (UC5.1.2E3)	0.09	0.02	0.04	0.06	0.26	0.01	0.06	0.1	0.09	0.019	0.232
Unidentified *Coriobacter* (CHKCI002)	0.09	0.08	0.04	0.09	0.02	0.19	0.07	0.02	0.08	0.012	0.371
Unidentified *Coriobacter* (CHKCI001)	0.07	0.08	0.05	0.13	0.11	0.04	0.07	0.06	0.05	0.009	0.830
Unidentified *Ruminococcus* (GCA.900066225)	0.06	0.07	0.02	0.06	0.13	0.07	0.08	0.03	0.08	0.011	0.264
Escherichia/Shigella	n.d.	0.02	0.13	0.13	n.d.	0.05	0.04	0.07	0.11	0.023	0.087
Unidentified *Christensenella* (R.7)	0.11	0.03	0.06	0.04	0.03	0.02	0.01	0.07	0.05	0.008	0.946
Anaeroplasma spp.	0.09	0.04	0.01	0.06	0.05	0.02	0.06	0.01	n.d.	0.028	0.035
Unidentified *Lachnospira* (UCG.010)	0.05	n.d.	n.d.	0.04	n.d.	0.05	0.08	0.06	0.01	0.011	0.335
Unidentified *Ruminococcus* (UCG.002)	n.d.	0.04	0.06	0.08	0.05	0.06	0.02	n.d.	0.02	0.013	0.591
Anaerofustis spp.	n.d.	0.02	0.09	0.08	0.01	n.d.	0.03	0.23	n.d.	0.009	0.198
Caproiciproducens spp.	0.04	0.05	n.d.	n.d.	0.07	n.d.	0.05	0.05	n.d.	0.006	0.508

Unidentified Lachnospira (NC2004)	n.d.	n.d.	0.09	0.16	0.02	0.01	0.02	0.02	n.d.	0.01	0.808
Candidatus_Soleaferrea spp.	n.d.	0.02	0.01	0.01	n.d.	n.d.	0.09	0.1	0.03	0.01	0.800
Ruminiclostridium spp.	n.d.	0.01	0.04	0.03	0.02	0.03	0.01	0.06	0.05	0.009	0.497
Hydrogenoanaerobacterium spp.	n.d.	0.08	0.1	n.d.	0.03	0.01	0.01	0.02	n.d.	1.256	0.673
Unidentified *Ruminococcus* (UCG.008)	n.d.	0.01	0.01	0.01	0.03	0.08	0.03	n.d.	0.06	0.859	0.262
Unidentified *Ruminococcus* (UCG.004)	0.02	n.d.	n.d.	0.03	0.01	0.03	0.03	0.03	0.1	1.535	0.195
Romboutsia spp.	0.01	n.d.	0.02	0.02	0.01	0.03	0.01	0.03	0.02	0.651	0.670
Intestinibacter spp.	0.04	0.01	0.04	n.d.	0.04	n.d.	n.d.	0.01	0.02	0.575	0.900

n.d. = not detected

[1] = Kruskal-Wallis Test; superscripts denote significant differences within a row (Mann-Whitney U Test, $p \leq 0.05$); Capital letters denote trends for significant difference (Mann-Whitney U Test, $p \leq 0.1$)

Table S8: Ecological indices of the intestinal microbiota in the crop of 13-day-old broiler chicken fed different probiotics and phytobiotics

	Control	LS1	LA73	Formulation C	Formulation L	LS1 & Formulation C	LS1 & Formulation L	LA73 & Formulation C	LA73 & Formulation L	Pooled SEM	p-value[1]
Richness	12.3	11.2	11.6	12.6	12.0	10.0	9.8	9.8	8.0	0.47	0.229
Shannon Index	1.09^{b}	0.94^{b}	1.16^{b}	0.74^{a}	1.13^{b}	0.84^{a}	0.72^{a}	1.12^{b}	1.11^{b}	0.04	0.015
Evenness	0.446^{b}	0.385^{a}	0.485^{b}	0.295^{a}	0.480^{b}	0.388^{a}	0.334^{a}	0.494^{b}	0.535^{b}	0.02	0.033

[1] = Kruskal-Wallis Test; superscripts denote significant differences within a row (Mann-Whitney U Test, $p \leq 0.05$)

Table S9: Ecological indices of the intestinal microbiota in the caecum of 13-day-old broiler chicken fed different probiotics and phytobiotics

	Control	LS1	LA73	Formulation C	Formulation L	LS1 & Formulation C	LS1 & Formulation L	LA73 & Formulation C	LA73 & Formulation L	Pooled SEM	p-value[1]
Richness	40.4	41.6	45.8	44.0	40.6	43.8	45.0	43.4	43.6	0.84	0.858
Shannon Index	2.19	2.20	2.28	2.27	2.36	2.32	2.25	2.29	2.20	0.03	0.838

Evenness	0.594	0.591	0.598	0.601	0.639	0.615	0.593	0.608	0.585	0.01	0.568

[1] = Kruskal-Wallis Test; superscripts denote significant differences within a row (Mann-Whitney U Test, $p \leq 0.05$)

Table S10: 16S rDNA copy numbers of *Clostridium perfringens* in the intestinal tract of 13-day-old broiler chicken fed different probiotics and phytobiotics [log copy number 16S rDNA]

	Crop	N[1]	Caecum	N[1]
Control	4.7 (±1.0)	3	5.6 (±1.4)	5
LS1	3.6 (±0.9)	3	4.2 (±0.9)	2
LA73	n.d.	0	3.5	1
Formulation C	4.5 (±0.7)	2	3.9 (±0.8)	2
Formulation L	3.8	1	3.5	1
LS1 & Formulation C	3.4 (±0.5)	2	3.8	1
LS1 & Formulation L	n.d.	1	3.3	1
LA73 & Formulation C	4.6	1	3.6	1
LA73 & Formulation L	3.9	1	3.9	1

[1] = number of positive samples

n.d. = not detected
